# Orthogonality, Lommel integrals and cross product zeros of linear combinations of Bessel functions

**DOI:** 10.1186/s40064-015-1142-0

**Published:** 2015-08-04

**Authors:** Christian H Ziener, Felix T Kurz, Lukas R Buschle, Thomas Kampf

**Affiliations:** Department of Radiology, German Cancer Research Center-DKFZ, Im Neuenheimer Feld 280, 69120 Heidelberg, Germany; Division of Neuroradiology, Heidelberg University, Im Neuenheimer Feld 400, 69120 Heidelberg, Germany; Department of Experimental Physics 5, University of Würzburg, Am Hubland, 97074 Würzburg, Germany

**Keywords:** Bessel function, Linear combination, Integral

## Abstract

The cylindrical Bessel differential equation and the spherical Bessel differential equation in the interval $R \le r \le \gamma R$ with Neumann boundary conditions are considered. The eigenfunctions are linear combinations of the Bessel function $\Phi _{n,\nu }(r)=Y_{\nu }^{\prime }(\lambda _{n,\nu }) J_{\nu }(\lambda _{n,\nu } r/R)-J_{\nu }^{\prime }(\lambda _{n,\nu }) Y_{\nu }(\lambda _{n,\nu } r/R)$ or linear combinations of the spherical Bessel functions $\psi _{m,\nu }(r)=y_{\nu }^{\prime }(\lambda _{m,\nu }) j_{\nu }(\lambda _{m,\nu } r/R)-j_{\nu }^{\prime }(\lambda _{m,\nu }) y_{\nu }(\lambda _{m,\nu } r/R)$. The orthogonality relations with analytical expressions for the normalization constant are given. Explicit expressions for the Lommel integrals in terms of Lommel functions are derived. The cross product zeros $Y_{\nu }^{\prime }(\lambda _{n,\nu }) J_{\nu }^{\prime }(\gamma \lambda _{n,\nu })-J_{\nu }^{\prime }(\lambda _{n,\nu }) Y_{\nu }^{\prime }(\gamma \lambda _{n,\nu }) = 0$ and $y_{\nu }^{\prime }(\lambda _{m,\nu }) j_{\nu }^{\prime }(\gamma \lambda _{m,\nu })-j_{\nu }^{\prime }(\lambda _{m,\nu }) y_{\nu }^{\prime }(\gamma \lambda _{m,\nu }) = 0$ are considered in the complex plane for real as well as complex values of the index $\nu$ and approximations for the exceptional zero $\lambda _{1,\nu }$ are obtained. A numerical scheme based on the discretization of the two-dimensional and three-dimensional Laplace operator with Neumann boundary conditions is presented. Explicit representations of the radial part of the Laplace operator in form of a tridiagonal matrix allow the simple computation of the cross product zeros.

## Background

Cylindrical Bessel functions and spherical Bessel functions are widely used in mathematics (Watson 1966), physics (Gray and Mathews 1895) and engineering science (McLachlan 1941) to analyze boundary value problems in cylindrical or spherical geometries. Using integral transform techniques or eigenfunction expansions, a second order differential equation remains that can be transformed into a cylindrical Bessel differential equation or a spherical Bessel differential equation. The general solution can be given in terms of a linear combination of cylindrical Bessel functions *J* and *Y* or, in the three-dimensional case, in terms of a linear combination of spherical Bessel functions *j* and *y*. These eigenfunctions are orthogonal to each other and the corresponding eigenvalues can be found as the zeros of a specific cross product consisting of cylindrical and spherical Bessel functions, respectively. Furthermore, to calculate expansion coefficients of an eigenfunction expansion, it is helpful to evaluate Lommel integrals, which contain the eigenfunctions and a power function.

Cylindrical Bessel functions and their properties are well described, as for example in the textbook of Watson (1966) where orthogonality relations and Lommel integrals are analyzed in detail. However, for a linear combination of spherical Bessel functions such orthogonality relations or Lommel integrals are not given explicitly, although they occur in many problems of diffusion or heat conduction in spherical geometries (for a review, see Carslaw and Jaeger 1959). This is mainly due to the fact that spherical Bessel functions with an integer index can be expressed in terms of trigonometric functions, and, thus, the remaining orthogonality relations and Lommel integrals can be given explicitly. Yet, the case for arbitrary complex-valued indices of cylindrical and spherical Bessel functions has not been analyzed and there are no generally valid expressions for the orthogonality relations and the Lommel integral.

Moreover, the cross product zeros of Bessel functions that provide the eigenvalues in the case of an eigenfunction expansion are highly relevant for inverse eigenvalue problems and go back to the famous question ”Can one hear the shape of a drum?” asked by Kac (1966). Cross product zeros were first analyzed by McMahon (1894) who derived an expression for large zeros. In addition, Cochran examined the asymptotic nature and analyticity of cross product Bessel functions (Cochran 1964, 1966a, b). Recurrence relations for the Bessel function cross products are given by Goodwin (1949). But, until now, the cross products of cylindrical Bessel functions or spherical Bessel functions for an arbitrary complex index of the Bessel function have not yet been discussed in depth, although its application in physics becomes increasingly important, e.g. in optics or quantum mechanics, where non-hermitean potentials are involved.

In this work, the cylindrical and spherical Bessel differential equation, respectively, are considered in the radial interval $R \le r \le \gamma R$ where $\gamma \ge 1$. In "Preliminary facts", we investigate the orthogonality relation between eigenfunctions and the Lommel integral for a linear combination of cylindrical Bessel functions *J* and *Y*. In an analogous procedure, general expressions for the orthogonality relation and Lommel integral for a linear combination of spherical Bessel functions *j* and *y* are derived by using similarity relations between cylindrical and spherical Bessel functions. In "Cylindrical Bessel functions", the cylindrical Bessel differential equation $R^2 [\nu ^2/r^2 - \Delta _r] \Phi _{n,\nu }(r) = \lambda _{n,\nu }^2 \Phi _{n,\nu }(r)$ is considered for Neumann boundary conditions on both ends of the radial interval. Explicit expressions for the orthogonality relation and Lommel integral of the according eigenfunctions $\Phi _{n,\nu }(r)=Y_{\nu }^{\prime }(\lambda _{n,\nu }) J_{\nu }(\lambda _{n,\nu } r/R)-J_{\nu }^{\prime }(\lambda _{n,\nu }) Y_{\nu }(\lambda _{n,\nu } r/R)$ are given where the eigenvalues $\lambda _{n,\nu }$ are determined by the cross product zeros $Y_{\nu }^{\prime }(\lambda _{n,\nu }) J_{\nu }^{\prime }(\gamma \lambda _{n,\nu })-J_{\nu }^{\prime }(\lambda _{n,\nu }) Y_{\nu }^{\prime }(\gamma \lambda _{n,\nu }) = 0$. To analyze cross product zeros for a complex valued index $\nu$, the function $f_{\nu }(\lambda ) = Y_{\nu }^{\prime }(\lambda ) J_{\nu }^{\prime } (\gamma \lambda ) - J_{\nu }^{\prime }(\lambda ) Y_{\nu }^{\prime } (\gamma \lambda )$ is considered in the complex $\lambda$-plane and symmetry relations of the function $f_{\nu }(\lambda )$ are provided. The first exceptional zero is approximated by a Taylor expansion of the function $f_{\nu }(\lambda )$. A discretization scheme for the radial part of two-dimensional Laplace-operator with Neumann boundary conditions is proposed and given in terms of a tridiagonal matrix. Consequently, the cross-product zeros can be obtained by solving a simple matrix eigenvalue problem. In "Spherical Bessel functions", similar results are derived for the spherical Bessel differential equation $R^2 [\nu [\nu +1]/r^2 - \Delta _r] \psi _{m,\nu }(r) = \lambda _{m,\nu }^2 \psi _{m,\nu }(r)$ and the corresponding eigenfunctions $\psi _{m,\nu }(r)=y_{\nu }^{\prime }(\lambda _{m,\nu }) j_{\nu }(\lambda _{m,\nu } r/R)-j_{\nu }^{\prime }(\lambda _{m,\nu }) y_{\nu }(\lambda _{m,\nu } r/R)$. In "Numerical implementation", numerical algorithms are provided that correctly implement discretization schemes for the radial part of the two-dimensional and three-dimensional Laplace-operator and that allow a direct determination of cross product zeros for both cylindrical and spherical Bessel functions. Applications to the diffusion process around dipole fields are described in "Application to the diffusion process around dipole fields". A summary of the results and conclusions are given in "Summary and conclusions".

## Preliminary facts

### Cylindrical Bessel functions

For a linear combination of Bessel functions

1 $$\begin{aligned} \Psi _{\nu }(z)=AJ_{\nu }(z)+BY_{\nu }(z) \end{aligned}$$

the following orthogonality relation holds [see Eq. (11.4.2) on page 485 in Abramowitz and Stegun (1972) or Eq. (11) on page 135 in Watson (1966) or Eqs. (19) and (20) on page 97 in McLachlan (1941)]:

2 $$\begin{aligned} \int \limits _a^b \mathrm {d}t \ t\, \Psi _{n,\nu }(\lambda _{n,\nu } t) \Psi _{n^{\prime },\nu } (\lambda _{n^{\prime },\nu } t) = N_{n,\nu } \delta _{nn^{\prime }} \end{aligned}$$

where the index $\nu$ denotes the order of the cylindrical Bessel function and *n* numerates the eigenvalues $\lambda _{n,\nu }$ which obey the boundary conditions

3 $$\begin{aligned} \Psi _{n,\nu }^{\prime } (\lambda _{n,\nu } a)&= \alpha \Psi _{n,\nu }(\lambda _{n,\nu } a) \end{aligned}$$

4 $$\begin{aligned} \Psi _{n,\nu }^{\prime } (\lambda _{n,\nu } b)&= \beta \Psi _{n,\nu }(\lambda _{n,\nu } b) \,. \end{aligned}$$

The normalization constant is

5 $$\begin{aligned} N_{n,\nu } = \left\{ \frac{t^2}{2} \left[ 1-\frac{\nu ^2}{\lambda _{n,\nu }^2 t^2} \right] \left[ \Psi _{n,\nu }(\lambda _{n,\nu } t)\right] ^2 + \frac{t^2}{2} \left[ \Psi _{n,\nu }^{\prime }(\lambda _{n,\nu } t)\right] ^2 \right\} _a^b \,. \end{aligned}$$

The Lommel integral involving the linear combination of Bessel functions and a power function is given in the following form:

6 $$\begin{aligned} \int \limits _a^b \mathrm {d}z \, z^{\kappa } \Psi _{n,\nu }(z) = \left\{ z \left[ \Psi _{n,\nu }(z) S_{\kappa ,\nu }^{\prime }(z) - \Psi _{n,\nu }^{\prime }(z) S_{\kappa ,\nu }(z) \right] \right\} _a^b \end{aligned}$$

which can be given in terms of the function $\Psi _{n,\nu }(z)$ and its derivative at the boundaries [see Eq. (5) on page 350 in paragraph 10.74 in Watson (1966) in combination with Eq. (3) on page 83 in paragraph 3.9 in Watson (1966)]. It can be evaluated using the Lommel functions

7 $$\begin{aligned} S_{\kappa ,\nu }(z)&=s_{\kappa ,\nu }(z) \nonumber \\&\quad + 2^{\kappa - 1} \Gamma \left( \frac{1+\kappa -\nu }{2} \right) \Gamma \left( \frac{1+\kappa +\nu }{2} \right) \left[ \sin \left( \pi \frac{\kappa -\nu }{2} \right) J_{\nu }(z) - \cos \left( \pi \frac{\kappa -\nu }{2} \right) Y_{\nu }(z) \right] \end{aligned}$$

8 $$\begin{aligned} s_{\kappa ,\nu }(z)&=\frac{z^{\kappa +1}}{[\kappa +1]^2-\nu ^2} \, _1F_2\left( 1;\frac{\kappa -\nu +3}{2},\frac{\kappa +\nu +3}{2};-\frac{z^2}{4} \right) \,. \end{aligned}$$

The Lommel functions obey the following recurrence relations (Watson 1966; Babister 1967; Ziener and Schlemmer 2013):

9 $$\begin{aligned} S_{\kappa +2,\nu }(z)&=z^{\kappa +1}-\left[ [\kappa + 1]^2 -\nu ^2 \right] S_{\kappa ,\nu }(z)\end{aligned}$$

10 $$\begin{aligned} S_{\kappa ,\nu }^{\prime }(z) + \frac{\nu }{z} S_{\kappa ,\nu }(z)&= \left[ \kappa + \nu - 1 \right] S_{\kappa -1,\nu -1}(z) \end{aligned}$$

11 $$\begin{aligned} S_{\kappa ,\nu }^{\prime }(z) - \frac{\nu }{z} S_{\kappa ,\nu }(z)&= \left[ \kappa - \nu - 1 \right] S_{\kappa -1,\nu +1}(z) \,. \end{aligned}$$

For the special cases $\kappa = 1$ and $\nu =2m$ as well as $\kappa = 0$ and $\nu =2m+1$, the Lommel functions can be written in terms of Neumann polynomials $O_m$:

12 $$\begin{aligned} S_{1,2m}(z)&=zO_{2m}(z) \end{aligned}$$

13 $$\begin{aligned} S_{0,2m+1}(z)&=\frac{z}{2m+1} O_{2m+1}(z) \end{aligned}$$

where

14 $$\begin{aligned} O_0(z)&=\frac{1}{z} \end{aligned}$$

15 $$\begin{aligned} O_1(z)&=\frac{1}{z^2}\end{aligned}$$

16 $$\begin{aligned} O_2(z)&=\frac{1}{z}+\frac{4}{z^3}\nonumber \\&\;\;\vdots \end{aligned}$$

[see page 274 in Watson (1966)]. In a similar way the special cases $\kappa = -1$ and $\nu =2m$ as well as $\kappa = 0$ and $\nu =2m+1$ can be written in terms of Schläfli polynomials $S_m$ as:

17 $$\begin{aligned} S_{-1,2m}(z)&=\frac{1}{4m}S_{2m}(z)\end{aligned}$$

18 $$\begin{aligned} S_{0,2m+1}(z)&=\frac{1}{2} S_{2m+1}(z) \end{aligned}$$

where

19 $$\begin{aligned} S_0(z)&=0 \end{aligned}$$

20 $$\begin{aligned} S_1(z)&=\frac{2}{z}\end{aligned}$$

21 $$\begin{aligned} S_2(z)&=\frac{4}{z^2}\nonumber \\&\;\;\vdots \end{aligned}$$

[see Eq. (2) on page 285 and on page 286 in paragraph 9.3 in Watson (1966)]. The exceptional case $S_{-1,0}(z)$ is treated in Watson (1966) and Glasser (2010).

The most general case in which Lommel functions $S_{\kappa ,\nu }$ can be expressed in terms of power functions occurs when the sum of the indices $\kappa + \nu = 2 p + 1$ is an odd integer [see Eq. (8) in section 10.74 on page 351 in Watson (1966)]:

22 $$\begin{aligned} S_{\gamma ,2p+1-\gamma } (z) = \frac{p!}{2^{1-\gamma } \Gamma (p+1-\gamma )} \frac{z^{\gamma }}{2p+1-\gamma } A_{2p,1-\gamma }(z) \end{aligned}$$

where the Gegenbauer polynomials $A_{n,\nu }$ are defined as [see Eq. (1) in section 9.2 on page 283 in Watson (1966)]:

23 $$\begin{aligned} A_{n,\nu }(z) = 2^{\nu +n} \frac{\nu +n}{z^{n+1}} \sum _{m=0}^{\lfloor \frac{n}{2} \rfloor } \frac{\Gamma (\nu +n-m)}{m!} \left[ \frac{z}{2}\right] ^{2m} \,. \end{aligned}$$

From this definition it is easy to obtain the relation

24 $$\begin{aligned} A_{2p,1-\gamma }(z)=zA_{2p+1,-\gamma }(z) \,, \end{aligned}$$

and, further, to give the following explicit expressions:

25 $$\begin{aligned} A_{0,\nu }(z)&= \Gamma (\nu +1) \frac{2^{\nu }}{z}\end{aligned}$$

26 $$\begin{aligned} A_{1,\nu }(z)&= \Gamma (\nu +2) \frac{2^{\nu +1}}{z^2}\end{aligned}$$

27 $$\begin{aligned} A_{2,\nu }(z)&= \Gamma (\nu +3) \frac{4+4\nu +z^2}{\nu +1} \frac{2^{\nu }}{z^3}\nonumber \\&\;\;\vdots \end{aligned}$$

The term for $A_{0,\nu }(z)$ given in Eq. (25) is in agreement with the results of Watson [see Eq. (7) in section 9.2 on page 283 in Watson (1966)]. Other special cases of the Lommel functions can be found in section 3.10.2 on pages 111–113 in Magnus et al. (1966).

### Spherical Bessel functions

To obtain similar relations for spherical Bessel functions, it is advantageous to consider the linear combination

28 $$\begin{aligned} \psi _{\mu }(z)=A j_{\mu }(z)+B y_{\mu }(z) \end{aligned}$$

and to use the following general relation between cylindrical Bessel functions and spherical Bessel functions:

29 $$\begin{aligned} \Psi _{\mu +\frac{1}{2}}(z)&= \sqrt{\frac{2z}{\pi }} \psi _{\mu }(z) \end{aligned}$$

30 $$\begin{aligned} \Psi _{\mu +\frac{1}{2}}^{\prime }(z)&= \frac{1}{\sqrt{2\pi z}} \left[ \psi _{\mu }(z) + 2z\psi _{\mu }^{\prime }(z) \right] \,. \end{aligned}$$

Introducing the relations (29) and (30) into the orthogonality relation (2) of cylindrical Bessel functions, the following orthogonality relation for spherical Bessel functions can be obtained:

31 $$\begin{aligned} \int \limits _a^b \mathrm {d}t \, t^2 \psi _{m,\mu }(\lambda _{m,\mu } t) \psi _{m^{\prime },\mu }(\lambda _{m^{\prime },\mu } t) = N_{m,\mu } \delta _{m m^{\prime }}\, , \end{aligned}$$

where the same notation as in the case of cylindrical Bessel functions is used, i.e. the index $\mu$ denotes the order of the spherical Bessel functions and the index *m* enumerates the eigenvalues $\lambda _{m,\mu }$ which obey the boundary condition

32 $$\begin{aligned} \psi _{m,\mu }^{\prime } (\lambda _{m,\mu } a)&= \gamma \psi _{m,\mu }(\lambda _{m,\mu } a) \end{aligned}$$

33 $$\begin{aligned} \psi _{m,\mu }^{\prime } (\lambda _{m,\mu } b)&= \delta \psi _{m,\mu }(\lambda _{m,\mu } b) \,. \end{aligned}$$

The corresponding normalization constant is given by

34 $$\begin{aligned} N_{m,\mu } &= \left\{ \frac{t}{2} \left[ t^2 - \mu \frac{\mu + 1}{\lambda _{m,\mu }^2}\right] \left[ \psi _{m,\mu }(\lambda _{m,\mu } t) \right] ^2 + \frac{t^2}{2\lambda _{m,\mu }} \psi _{m,\mu }(\lambda _{m,\mu } t) \psi _{m,\mu }^{\prime }(\lambda _{m,\mu } t)\right. \\ & \left. \quad + \frac{t^3}{2}\left[ \psi _{m,\mu }^{\prime }(\lambda _{m,\mu } t) \right] ^2 \right\} _a^b \,. \end{aligned}$$

The Lommel integral can be evaluated by using Eqs. (29) and (30):

35 $$\begin{aligned} \int \limits _a^b \!\mathrm {d}z \, z^{\kappa } \psi _{m,\mu }(z) &= \left\{ \sqrt{z} \psi _{m,\mu }(z) \left[ z S_{\kappa -\frac{1}{2},\mu +\frac{1}{2}}^{\prime } (z) - \frac{1}{2} S_{\kappa -\frac{1}{2},\mu +\frac{1}{2}} (z) \right] \right. \\ & \left. \quad- z \sqrt{z} \psi _{m,\mu }^{\prime } (z) S_{\kappa -\frac{1}{2},\mu +\frac{1}{2}} (z) \right\} _a^b \,. \end{aligned}$$

## Cylindrical Bessel functions

### Cross-product zeros

The cylindrical Bessel differential equation

36 $$\begin{aligned} \left[ \frac{\nu ^2}{r^2} - \Delta _r \right] \Phi _{n,\nu } (r) = \frac{\lambda _{n,\nu }^2}{R^2} \Phi _{n,\nu } (r) \end{aligned}$$

with the radial part of two-dimensional Laplace operator $\Delta _r = \partial _{rr}+r^{-1} \partial _r$ inside an annular ring $R \le r \le \gamma R$ is considered. Reflecting boundary conditions at $r=R$ and $r=\gamma R$ are assumed:

37 $$\begin{aligned}&\left. \frac{\partial }{\partial r} \Phi _{n,\nu }(r) \right| _{r=R} = 0 \end{aligned}$$

38 $$\begin{aligned}&\left. \frac{\partial }{\partial r} \Phi _{n,\nu }(r) \right| _{r=\gamma R} = 0 \,. \end{aligned}$$

The respective eigenfunctions are given by

39 $$\begin{aligned} \Phi _{n,\nu }(r)= Y_{\nu }^{\prime }(\lambda _{n,\nu }) J_{\nu } \left( \lambda _{n,\nu } \frac{r}{R} \right) - J_{\nu }^{\prime }(\lambda _{n,\nu }) Y_{\nu } \left( \lambda _{n,\nu } \frac{r}{R} \right) \,, \end{aligned}$$

which obviously obey the reflecting boundary condition at $r=R$.

Reflecting boundary conditions at the outer boundary at $r=\gamma R$ lead to the conditions

40 $$\begin{aligned} f_{\nu }(\lambda _{n,\nu })&= 0 \end{aligned}$$

41 $$\begin{aligned} f_{\nu }(\lambda )&= Y_{\nu }^{\prime }(\lambda ) J_{\nu }^{\prime } (\gamma \lambda ) - J_{\nu }^{\prime }(\lambda ) Y_{\nu }^{\prime } (\gamma \lambda ) \,, \end{aligned}$$

that have to be evaluated numerically. The function $f_{\nu }(\lambda )$ possesses the following symmetry properties

42 $$\begin{aligned} f_{\nu }(+\lambda )&=f_{\nu }(-\lambda ) \end{aligned}$$

43 $$\begin{aligned} f_{+\nu }(\lambda )&=f_{-\nu }(\lambda ) \end{aligned}$$

44 $$\begin{aligned} f_{\nu ^{*}}(\lambda )&=f_{\nu }^{*}(\lambda ^{*}) \end{aligned}$$

that translates to the properties of eigenvalues

45 $$\begin{aligned} \lambda _{n,+\nu }&=\lambda _{n,-\nu } \end{aligned}$$

46 $$\begin{aligned} \lambda _{n,\nu ^{*}}&=\lambda _{n,\nu }^{*} \end{aligned}$$

and eigenfunctions

47 $$\begin{aligned} \Phi _{n,+\nu }(r)&=\Phi _{n,-\nu }(r) \end{aligned}$$

48 $$\begin{aligned} \Phi _{n,\nu ^{*}}(r)&=\Phi _{n,\nu }^{*}(r) \,. \end{aligned}$$

For real values of the Bessel function index $\nu$ the function $f_{\nu }(\lambda )$ always takes real values and the eigenvalues $\lambda _{n,\nu }$ are real. The respective eigenvalues $\lambda _{n,\nu }$ are tabulated (Bauer 1964; Bridge and Angrist 1962; Truell 1943) or they can be found by using numerical routines (Sorolla et al. 2013) and empirical approximations (Laslett and Lewish 1962). The computer algebra system MATHEMATICA^®^ (Wolfram Research, Inc., Champaign, IL, USA, Wolfram 1999) provides the command

49 $$\begin{aligned} \lambda _{n,\nu }=\mathtt{BesselJPrimeYPrimeJPrimeYPrimeZeros[\nu , \gamma , n][[n]]} \end{aligned}$$

for numerical computation of the eigenvalues. However, these standard numerical routines based on search algorithms for zeros can run into problems for inappropriate initial conditions of the algorithm. Consequently eigenvalues can be missed or found multiple. Furthermore, for complex values of the index of the Bessel functions, these numerical routines often fail. To circumvent these problems, an algorithm based on solving the corresponding original eigenvalue problem will be introduced in "Numerical implementation".

**Fig. 1:**
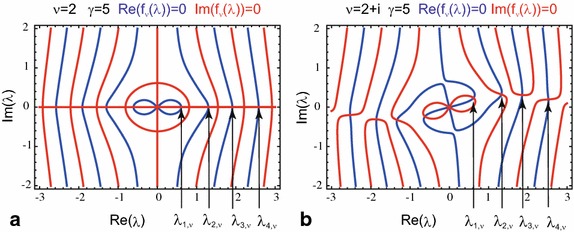
Contour plot of the function $f_{\nu }(\lambda )$ in the complex $\lambda$-plane. **a** On the *blue lines*, the real part of the function $f_{\nu }(\lambda )$ vanishes, i.e. $\text {Re}(f_{\nu }(\lambda ))=0$, and, on the *red lines*, the imaginary part of the function $f_{\nu }(\lambda )$ vanishes, i.e. $\text {Im}(f_{\nu }(\lambda ))=0$. The eigenvalues are located at the intersection points of the *blue lines* and *red lines*. For a real index (shown for $\nu =2$ and $\gamma = 5$ as an example), all intersection points are located on the real axis. **b** For a complex index (examplarily for $\nu =2+\mathrm {i}$ and $\gamma =5$), the contour plots become more sophisticated and the intersection points are displaced into the complex plane.

For complex values of the Bessel function index $\nu$, however, it is helpful to consider the complex valued function $f_{\nu }(\lambda )$ in the complex $\lambda$-plane as shown in Fig. 1. Similar considerations are performed in Figure 2 in Ziener et al. (2012).

For small values of the parameter $\lambda$, the function $f_{\nu }(\lambda )$ given in Eq. (41) can be approximated by

50 $$\begin{aligned} f_{\nu }(\lambda ) \approx \frac{\nu \left[ \gamma ^{2\nu } -1\right] }{\pi \lambda ^2 \gamma ^{1+\nu }} + \frac{\left[ \nu ^2+\nu -2 \right] \left[ 1-\gamma ^2 \right] \left[ 1+\gamma ^{2\nu } \right] -2\nu \left[ \gamma ^{2\nu }-\gamma ^2\right] }{4\pi \left[ \nu ^2-1\right] \gamma ^{1+\nu }} \end{aligned}$$

and the first zero of this approximated function is

51 $$\begin{aligned} \lambda _{1,\nu } \approx \sqrt{\frac{4\nu \left[ 1-\nu ^2 \right] \left[ \gamma ^{2\nu }-1 \right] }{\left[ \nu ^2+\nu -2 \right] \left[ 1-\gamma ^2 \right] \left[ 1+\gamma ^{2\nu } \right] -2\nu \left[ \gamma ^{2\nu }-\gamma ^2\right] }} \,. \end{aligned}$$

In the limit $\gamma \rightarrow 1$, the first eigenvalue tends to

52 $$\begin{aligned} \lim _{\gamma \rightarrow 1} \lambda _{1,\nu } = \nu \,. \end{aligned}$$

In addition, for small values of the index $\nu$, the first eigenvalue can be obtained from a Taylor expansion:

53 $$\begin{aligned} \lambda _{1,\nu } \approx \nu \sqrt{\frac{2 \ln (\gamma )}{\gamma ^2 - 1}} \,. \end{aligned}$$

In the same limit, Gottlieb derived [see Eq. (A.5) in Gottlieb (1985)]:

54 $$\begin{aligned} \lambda _{1,\nu } \approx \nu \left[ 1-\frac{1}{2} [\gamma -1] + \frac{7}{24} [\gamma -1]^2 \right] \end{aligned}$$

and Grebenkov obtained [see Eq. (30) in Grebenkov (2007)]:

55 $$\begin{aligned} \lambda _{1,\nu } \approx \nu \sqrt{2-\gamma +\frac{5}{6}[1-\gamma ]^2 + \frac{2}{3} [1-\gamma ]^3 - \frac{\nu ^2-16}{30} [1-\gamma ]^4} \,. \end{aligned}$$

Additionally, Buchholz found [see Eq. (10) on page 363 in section 3.2. in Buchholz (1949) or Cochran (1964)]:

56 $$\begin{aligned} \lambda _{1,\nu } \approx \frac{\nu }{\sqrt{\gamma }\left[ 1+\frac{[\gamma -1]^2}{12\gamma }+\frac{[8\nu ^2-3][\gamma -1]^4}{480\gamma ^2} - \frac{[144 \nu ^2 - 103][\gamma -1]^6}{120960 \gamma ^3}\right] } \,. \end{aligned}$$

**Fig. 2:**
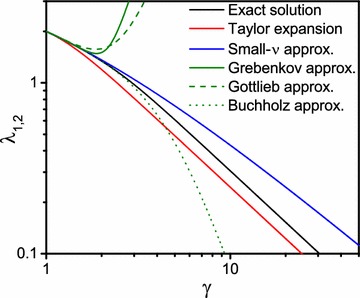
The exact numerical solution of the first ($n = 1$) zero $\lambda _{1,\nu }$, exemplary for $\nu = 2$ [*black solid line* obtained from Eq. (40)] in comparison with approximate solutions (*red solid line* obtained from Eq. (51) and blue solid line obtained from Eq. (53) for small values of the index $\nu$) and results from Grebenkov [*green solid line* obtained from Eq. (55)], Gottlieb [*green dashed line* obtained from Eq. (54)], and Buchholz [*green dotted line* obtained from Eq. (56)].

A comparison of the different approximations and the exact numerical solution is shown in Fig. 2.

For $n \ge 2$, the eigenvalues can be found with the approximation of McMahon (1894), see also 9.5.28 and 9.5.31 on page 374 of Abramowitz and Stegun (1972):

57 $$\begin{aligned} \lambda _{n,\nu } \approx \pi \frac{n-1}{\gamma -1} + \frac{4\nu ^2+3}{8\pi \gamma } \frac{\gamma -1}{n-1} + \frac{[\gamma ^2 + \gamma + 1][16\nu ^4 + 184\nu ^2 -63] - 6\gamma [4\nu ^2 + 3]^2}{384 \pi ^3 \gamma ^3} \left[ \frac{\gamma -1}{n-1}\right] ^3\,. \end{aligned}$$

Please note, that in Abramowitz and Stegun (1972) the exceptional zero is taken into account. Hence, there is a shift in the labeling of the zeros. The correct labeling of the zeros for example is given in 10.21.50 and 10.21.52 of Olver et al. (2010). The approximation coincides with the result of Grebenkov given in Eq. (31) in Grebenkov (2007). Another approximation for $n \ge 2$ was found by Buchholz [see Eq. (4) on page 364 in section 3.3. in Buchholz (1949)]:

58 $$\begin{aligned} \lambda _{n,\nu } \approx \frac{n-1}{\gamma -1} \frac{\pi }{1-\frac{4\nu ^2+3}{8\gamma \pi ^2} \left[ \frac{\gamma -1}{n-1}\right] ^2+\frac{96\nu ^4-176\nu ^2+198}{256\gamma ^2\pi ^4} \left[ \frac{\gamma -1}{n-1}\right] ^4} \,. \end{aligned}$$

### Discretization

For complex values of the index $\nu$, it is cumbersome to find the respective eigenvalues in the complex plane. To avoid a global search, a simple numerical procedure in terms of a discretization scheme is introduced. For this, the interval $R \le r \le \gamma R$ is divided into *p* intervals with length *h* in the following way:

59 $$\begin{aligned} r_j = R+[j-1] h \quad \text {for} \quad j=1, \ldots ,p \quad \text {and} \quad h=R\frac{\gamma -1 }{p-1}\,. \end{aligned}$$

As shown in Ziener et al. (2009, 2015), the radial part of the two-dimensional Laplace operator can be written in the form

60 $$\begin{aligned} \hat{\Delta }_r =\textstyle {\frac{1}{h^2}} \left( \!\! \begin{array}{llllllll} -2\frac{r_{1\frac{1}{2}}}{r_1} &{}\! +2\frac{r_{1\frac{1}{2}}}{r_1}&{} \\ +\frac{r_{1\frac{1}{2}}}{r_2} &{}\! -2\frac{r_2}{r_2} &{}\! +\frac{r_{2\frac{1}{2}}}{r_2} \\ &{}\! +\frac{r_{2\frac{1}{2}}}{r_3} &{}\! -2\frac{r_3}{r_3} &{}\! +\frac{r_{3\frac{1}{2}}}{r_3} \\ &{} &{} \ddots &{} \ddots &{} \ddots \\ &{} &{} &{}\! +\frac{r_{p-2\frac{1}{2}}}{r_{p-2}} &{}\! -2\frac{r_{p-2}}{r_{p-2}} &{}\! +\frac{r_{p-1\frac{1}{2}}}{r_{p-2}} \\ &{} &{} &{} &{}\! +\frac{r_{p-1\frac{1}{2}}}{r_{p-1}} &{}\! -2\frac{r_{p-1}}{r_{p-1}} &{}\! +\frac{r_{p-\frac{1}{2}}}{r_{p-1}} \\ &{} &{} &{} &{} &{}\! +2\frac{r_{p-\frac{1}{2}}}{r_{p}} &{}\! -2\frac{r_{p-\frac{1}{2}}}{r_{p}} \end{array} \!\!\right) \,. \end{aligned}$$

Fractional indices are meant as $r_{j\frac{1}{2}} = r_{j+\frac{1}{2}} = R+\left[ j-\frac{1}{2}\right] h$ and $r_{p-1\frac{1}{2}} = R + \left[ p-\frac{5}{2}\right] h$. Additionally, the main diagonal of this discretized radial part of the Laplace operator is given by $-2/h^2$ except for the first and the last row. Furthermore, the sum of all elements in each row must vanish. Thus, the discretized form of the Bessel differential equation (36) can be expressed as an eigenvalue equation:

61 $$\begin{aligned} \left[ \nu ^2 \mathrm {diag} \left( \frac{1}{r_1^2}, \ldots , \frac{1}{r_p^2}\right) - \hat{\Delta }_r \right] \hat{\Phi }_{n,\nu } = \frac{\lambda _{n,\nu }^2}{R^2} \hat{\Phi }_{n,\nu } \end{aligned}$$

where the eigenvector

62 $$\begin{aligned} \hat{\Phi }_{n,\nu } = \left( \Phi _{n,\nu } (r_1), \ldots , \Phi _{n,\nu } (r_p) \right) ^{\mathrm {T}} \end{aligned}$$

contains the values of the discretized eigenfunction $\Phi _{n,\nu }(r_j)$.

### Orthogonality

Due to Abel’s identity of the Wronski-determinant, the eigenfunctions take the value

63 $$\begin{aligned} \Phi _{n,\nu }(R)=\frac{2}{\pi \lambda _{n,\nu }} \end{aligned}$$

at the inner boundary at $r=R$. At the outer boundary at $r=\gamma R$, the eigenfunctions can be evaluated using the Wronski-determinant (63) and the eigenvalue equation (41):

64 $$\begin{aligned} \Phi _{n,\nu }(\gamma R)&= Y_{\nu }^{\prime }(\lambda _{n,\nu }) J_{\nu }(\gamma \lambda _{n,\nu } ) - J_{\nu }^{\prime }(\lambda _{n,\nu }) Y_{\nu } ( \gamma \lambda _{n,\nu } ) \end{aligned}$$

65 $$\begin{aligned}&= \frac{2}{\pi \gamma \lambda _{n,\nu }} \frac{J_{\nu }^{\prime }(\lambda _{n,\nu })}{J_{\nu }^{\prime }(\gamma \lambda _{n,\nu })} \end{aligned}$$

66 $$\begin{aligned}&= \frac{2}{\pi \gamma \lambda _{n,\nu }} \frac{Y_{\nu }^{\prime }(\lambda _{n,\nu })}{Y_{\nu }^{\prime }(\gamma \lambda _{n,\nu })}. \end{aligned}$$

The orthogonality relation of the eigenfunctions (39) can be obtained from the general expressions (2) and (5) by replacing the eigen functions with the expressions from Eqs. (63) and (64):

67 $$\begin{aligned}&\frac{1}{R^2} \int \limits _{R}^{\gamma R} \mathrm {d}r\, r \Phi _{n,\nu }(r) \Phi _{n^{\prime },\nu }(r) = N_{n,\nu } \delta _{nn^{\prime }}\end{aligned}$$

68 $$\begin{aligned}&N_{n,\nu } = \frac{2}{\pi ^2 \lambda _{n,\nu }^2} \left[ 1 - \frac{\nu ^2}{\gamma ^2 \lambda _{n,\nu }^2}\right] \left[ \frac{J_{\nu }^{\prime }(\lambda _{n,\nu })}{J_{\nu }^{\prime }(\gamma \lambda _{n,\nu })} \right] ^2 - \frac{2}{\pi ^2 \lambda _{n,\nu }^2} \left[ 1- \frac{\nu ^2}{\lambda _{n,\nu }^2} \right] \,. \end{aligned}$$

### Lommel integral

Applying eigenfunction $\Phi _{n,\nu }(r)$ from Eq. (39) to the general Lommel integral in Eq. (6), we arrive at

69 $$\begin{aligned} \frac{1}{R^{\alpha + 1 }} \int \limits _{R}^{\gamma R} \mathrm {d}r\, r^{\alpha } \Phi _{n,\nu }(r) = \frac{2}{\pi \lambda _{n,\nu }^{\alpha +1}} \left[ \frac{J_{\nu }^{\prime }(\lambda _{n,\nu })}{J_{\nu }^{\prime }(\gamma \lambda _{n,\nu })} S_{\alpha ,\nu }^{\prime } ( \gamma \lambda _{n,\nu } ) - S_{\alpha ,\nu }^{\prime } ( \lambda _{n,\nu } ) \right] , \end{aligned}$$

where Eqs. (63) and (64) were used. The special case $\alpha =1$ and $\nu =0$ can be evaluated using the relation Eq. (12) with the respective Neumann polynomial given in Eq. (14). Since the corresponding Lommel function takes the constant value $S_{1,0}(z)=1$, this special case unfolds as

70 $$\begin{aligned} \int \limits _{R}^{\gamma R} \mathrm {d}r\, r \Phi _{n,0}(r) = 0\,, \end{aligned}$$

i.e. the constant function 1 and the function $\Phi _{n,0}(r)$ are orthogonal in agreement with Thambynayagam (2011) (chapter 2.5, page 36). This result can be generalized by integrating the original cylindrical Bessel differential equation (36):

71 $$\begin{aligned} \int \limits _{R}^{\gamma R} \mathrm {d}r\, r \left[ \frac{\nu ^2}{r^2} - \frac{\lambda _{n,\nu }^2}{R^2} \right] \Phi _{n,\nu } (r) = 0 \,. \end{aligned}$$

## Spherical Bessel functions

### Cross-product zeros

The spherical Bessel differential equation

72 $$\begin{aligned} \left[ \frac{\nu [\nu +1]}{r^2} - \Delta _{r} \right] \psi _{m,\nu } (r) = \frac{\lambda _{m,\nu }^2}{R^2} \psi _{m,\nu } (r) \end{aligned}$$

with the radial part of the three-dimension Laplace operator $\Delta _{r} = \partial _{rr}+2 r^{-1} \partial _{r}$ inside a spherical shell $R \le r \le \gamma R$ is considered. Reflecting boundary conditions at $r=R$ and $r=\gamma R$ are assumed:

73 $$\begin{aligned}&\left. \frac{\partial }{\partial r} \psi _{m,\nu }(r) \right| _{r=R} = 0 \end{aligned}$$

74 $$\begin{aligned}&\left. \frac{\partial }{\partial r} \psi _{m,\nu }(r) \right| _{r=\gamma R} = 0 \,. \end{aligned}$$

The respective eigenfunctions are given by

75 $$\begin{aligned} \psi _{m,\nu }(r)= y_{\nu }^{\prime }(\lambda _{m,\nu }) j_{\nu } \left( \lambda _{m,\nu } \frac{r}{R} \right) - j_{\nu }^{\prime }(\lambda _{m,\nu }) y_{\nu } \left( \lambda _{m,\nu } \frac{r}{R} \right) \,, \end{aligned}$$

which already fulfill the reflecting boundary condition at $r=R$.

The reflecting boundary conditions at the outer boundary at $r = \gamma R$ lead to an equation for the determination of the eigenvalues:

76 $$\begin{aligned} g_{\nu }(\lambda _{m,\nu })&= 0 \,\,\,\,\,\,\text {where}\end{aligned}$$

77 $$\begin{aligned} g_{\nu }(\lambda )&= y_{\nu }^{\prime }(\lambda ) j_{\nu }^{\prime } (\gamma \lambda ) - j_{\nu }^{\prime }(\lambda ) y_{\nu }^{\prime } (\gamma \lambda ) \,. \end{aligned}$$

**Fig. 3:**
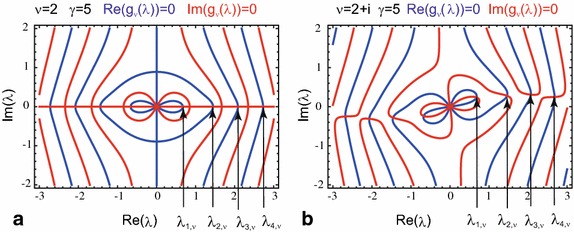
Contour plot of the function $g_{\nu }(\lambda )$ in the complex $\lambda$-plane. **a** As in Fig. 1, the real and the imaginary part of the function $g_{\nu }(\lambda )$ vanish on the blue lines and red lines, respectively, and the eigenvalues can be found at the intersection points of *blue* and *red lines*. For a real index (for $\nu =2$ and $\gamma =5$ as an example), all intersection points are located on the real axis. **b** For a complex index (exemplarily shown for $\nu =2+\mathrm {i}$ and $\gamma =5$), the geometry of the contour plots appears again more complicated as for real indices.

For real values of the Bessel function index ${\nu}$ the function $g_{\nu }(\lambda )$ always takes real values and the eigenvalues $\lambda _{m,\nu }$ are real. For complex values of the Bessel function index ${\nu}$ it is advantageous to consider the complex valued function $g_{\nu }(\lambda )$ in the complex $\lambda$-plane as visualized in Fig. 3. The function $g_{\nu }(\lambda )$ possesses similar symmetry properties as in the two-dimensional case:

78 $$\begin{aligned} g_{\nu }(+\lambda )&=-g_{\nu }(-\lambda ) \end{aligned}$$

79 $$\begin{aligned} g_{+\nu -\frac{1}{2}}(\lambda )&=g_{-\nu -\frac{1}{2}}(\lambda ) \end{aligned}$$

80 $$\begin{aligned} g_{\nu ^{*}}(\lambda )&=g_{\nu }^{*}(\lambda ^{*}) \,. \end{aligned}$$

This translates to the symmetry properties to eigenvalues

81 $$\begin{aligned} \lambda _{m,+\nu -\frac{1}{2}}&=\lambda _{m,-\nu -\frac{1}{2}} \end{aligned}$$

82 $$\begin{aligned} \lambda _{m,\nu ^{*}}&=\lambda _{m,\nu }^{*} \end{aligned}$$

and eigenfunctions

83 $$\begin{aligned} \psi _{m,+\nu -\frac{1}{2}}(r)&=\psi _{m,-\nu -\frac{1}{2}}(r) \end{aligned}$$

84 $$\begin{aligned} \psi _{m,\nu ^{*}}(r)&=\psi _{m,\nu }^{*}(r) \,. \end{aligned}$$

For small values of the parameter $\lambda$, the function $g_{\nu }(\lambda )$ given in Eq. (77) can be approximated by

85 $$\begin{aligned} g_{\nu }(\lambda )&=\frac{\nu }{\lambda ^3} \frac{\nu +1}{2 \nu +1} \frac{\gamma ^{2 \nu +1}-1}{ \gamma ^{2+\nu }}\end{aligned}$$

86 $$\begin{aligned}&+\frac{\left[ 2\nu ^3+5\nu ^2+\nu -2\right] \left[ 1-\gamma ^{2\nu +3}\right] +\nu \gamma ^2 \left[ 2\nu ^2+\nu -3\right] \left[ \gamma ^{2\nu -1}-1\right] }{2 \lambda [2 \nu +1] [4 \nu ^2+4 \nu -3] \gamma ^ {2+\nu }} \end{aligned}$$

and, thus, the first eigenvalue can be found at

87 $$\begin{aligned} \lambda _{1,\nu } \approx \sqrt{\frac{2\nu \left[ \nu +1\right] \left[ 4\nu ^2 +4\nu -3\right] \left[ 1-\gamma ^{2\nu +1}\right] }{\left[ 2\nu ^3+5\nu ^2+\nu -2\right] \left[ 1-\gamma ^{2\nu +3}\right] +\nu \gamma ^2 \left[ 2\nu ^2+\nu -3\right] \left[ \gamma ^{2\nu -1}-1\right] }} \,. \end{aligned}$$

In the limit $\gamma \rightarrow 1$, the first eigenvalue tends to

88 $$\begin{aligned} \lim _{\gamma \rightarrow 1} \lambda _{1,\nu } = \sqrt{\nu \left[ 1+\nu \right] } \,. \end{aligned}$$

For small values of the index $\nu$, the approximate value of first eigenvalue from Eq. (87) can be approximated further through a Taylor expansion:

89 $$\begin{aligned} \lambda _{1,\nu } \approx \sqrt{\frac{3\nu }{1+\gamma +\gamma ^2}} \,. \end{aligned}$$

In the same limit for small values of the parameter $\gamma$, Grebenkov found [see Eq. (34) in Grebenkov (2007)]:

90 $$\begin{aligned} \lambda _{1,\nu } \approx \sqrt{\nu [1+\nu ]} \sqrt{2-\gamma +\frac{2}{3} [1-\gamma ]^2 + \frac{1}{3}[1-\gamma ]^3 - \frac{3\nu [1+\nu ]-10}{90}[1-\gamma ]^4} \end{aligned}$$

and Gottlieb obtained [see Eq. (2.14) in Gottlieb (1985) or Eq. (A.10) in Gottlieb (1985)]:

91 $$\begin{aligned} \lambda _{1,\nu } \approx \sqrt{\nu [1+\nu ]}\left[ 1-\frac{1}{2}[\gamma -1]+\frac{5}{24}[\gamma -1]^2-\frac{1}{16}[\gamma -1]^3+\frac{5-32\nu [\nu +1]}{1920}[\gamma -1]^4 \right] \,. \end{aligned}$$

**Fig. 4:**
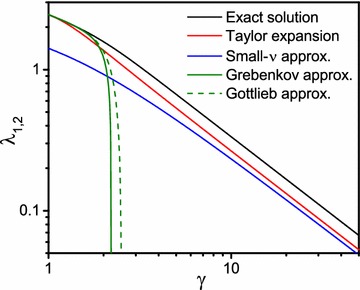
Exact numerical solution of the first eigenvalue $\lambda _{1,\nu }$ of Eq. (76) for $\nu =2$ (shown in the *black solid line*) and comparison with approximate solutions [*red solid line* from Eq. (87) and *blue solid line* from Eq. (89)) and results from Grebenkov (*green solid line* obtained from Eq. (91)] and Gottlieb [*green dashed line* obtained from Eq. (91)].

A comparison of the different approximations and the exact numerical solution is shown in Fig. 4.

For $m \ge 2$, we find with the approximation of McMahon (1894):

92 $$\begin{aligned} \lambda _{m,\nu } \approx \pi \frac{m-1}{\gamma -1} + \frac{\nu ^2+\nu +2}{2\pi \gamma } \frac{\gamma -1}{m-1} \end{aligned}$$

that is in agreement with Eq. (36) in Grebenkov (2007).

### Discretization

In analogy to the two-dimensional case, the radial interval $R \le r \le \gamma R$ can be discretized in the same form as given in Eq. (59):

93 $$\begin{aligned} r_j = R+[j-1] h \quad \text {for} \quad j=1, \ldots ,p \quad \text {and} \quad h=R\frac{\gamma -1 }{p-1}\,. \end{aligned}$$

As shown in Ziener et al. (2009), the radial part of the three-dimensional Laplace operator can be represented as

94 $$\begin{aligned} \hat{\Delta }_r =\textstyle {\frac{1}{h^2}} \left( \!\!\!\! \begin{array}{llllllll} -2 \frac{r_{1\frac{1}{2}}^2}{r_1^2} &{}\! +2 \frac{r_{1\frac{1}{2}}^2}{r_1^2}&{} \\ +\frac{r_{1\frac{1}{2}}^2}{r_2^2} &{}\! -\frac{r_{1\frac{1}{2}}^2+r_{2\frac{1}{2}}^2}{r_2^2} &{}\! + \frac{r_{2\frac{1}{2}}^2}{r_2^2} \\ &{}\! +\frac{r_{2\frac{1}{2}}^2}{r_3^2} &{}\! -\frac{r_{2\frac{1}{2}}^2+r_{3\frac{1}{2}}^2}{r_3^2} &{}\! +\frac{r_{3\frac{1}{2}}^2}{r_3^2} \\ &{} &{} \ddots &{} \ddots &{} \ddots \\ &{} &{} &{}\! +\frac{r_{p-2\frac{1}{2}}^2}{r_{p-2}^2} &{}\! -\frac{r_{p-2\frac{1}{2}}^2+r_{p-1\frac{1}{2}}^2}{r_{p-2}^2} &{}\! +\frac{r_{p-1\frac{1}{2}}^2}{r_{p-2}^2} \\ &{} &{} &{} &{}\! +\frac{r_{p-1\frac{1}{2}}^2}{r_{p-1}^2} &{}\! -\frac{r_{p-1\frac{1}{2}}^2+r_{p-\frac{1}{2}}^2}{r_{p-1}^2} &{}\! + \frac{r_{p-\frac{1}{2}}^2}{r_{p-1}^2} \\ &{} &{} &{} &{} &{}\! +2 \frac{r_{p-\frac{1}{2}}^2}{r_{p}^2} &{}\! -2 \frac{r_{p-\frac{1}{2}}^2}{r_{p}^2} \end{array} \!\!\!\!\right) \,. \end{aligned}$$

Thus, the discretized form of the spherical Bessel differential equation (72) can be written in the form of the eigenvalue equation:

95 $$\begin{aligned} \left[ \nu [\nu +1] \mathrm {diag} \left( \frac{1}{r_1^2}, \ldots , \frac{1}{r_p^2}\right) - \hat{\Delta }_r \right] \hat{\psi }_{m,\nu } = \frac{\lambda _{m,\nu }^2}{R^2} \hat{\psi }_{m,\nu } \end{aligned}$$

where the eigenvector

96 $$\begin{aligned} \hat{\psi }_{m,\nu } = \left( \psi _{m,\nu } (r_1), \ldots , \psi _{m,\nu } (r_p) \right) ^{\mathrm {T}} \end{aligned}$$

contains the values of the discretized eigenfunction $\psi _{m,\nu }(r_j)$.

### Orthogonality

Due to Abel’s identity of the Wronski-determinant, the eigenfunctions take the value

97 $$\begin{aligned} \psi _{m,\nu }(R)=\frac{1}{\lambda _{m,\nu }^2} \end{aligned}$$

at the inner boundary at $r=R$. The value of the eigenfunction at the outer boundary at $r=\gamma R$ can be evaluated using the Wronski-determinant (97) and the eigenvalue equation (77):

98 $$\begin{aligned} \psi _{m,\nu }(\gamma R)&= y_{\nu }^{\prime }(\lambda _{m,\nu }) j_{\nu }(\gamma \lambda _{m,\nu }) - j_{\nu }^{\prime }(\lambda _{m,\nu }) y_{\nu } ( \gamma \lambda _{m,\nu } ) \end{aligned}$$

99 $$\begin{aligned}&= \frac{1}{\gamma ^2 \lambda _{m,\nu }^2} \frac{j_{\nu }^{\prime }(\lambda _{m,\nu })}{j_{\nu }^{\prime }(\gamma \lambda _{m,\nu })} \end{aligned}$$

100 $$\begin{aligned}&= \frac{1}{\gamma ^2 \lambda _{m,\nu }^2} \frac{y_{\nu }^{\prime }(\lambda _{m,\nu })}{y_{\nu }^{\prime }(\gamma \lambda _{m,\nu })} \,. \end{aligned}$$

The orthogonality relation of the eigenfunctions (75) can be obtained from the general expressions (31) and (34) by replacing the respective eigenfunctions with the expressions in Eqs. (97) and (99) as in the case of cylindrical Bessel functions:

101 $$\begin{aligned}&\frac{1}{R^3} \int \limits _{R}^{\gamma R} \mathrm {d}r\, r^2 \psi _{m,\nu }(r) \psi _{m^{\prime },\nu }(r) = N_{m,\nu } \delta _{mm^{\prime }}\end{aligned}$$

102 $$\begin{aligned}&N_{m,\nu } = \frac{1}{2 \gamma \lambda _{m,\nu }^4} \left[ \frac{j_{\nu }^{\prime }(\lambda _{m,\nu })}{j_{\nu }^{\prime }(\gamma \lambda _{m,\nu })} \right] ^2 \left[ 1- \frac{\nu [\nu +1]}{\gamma ^2 \lambda _{m,\nu }^2}\right] + \frac{1}{2 \lambda _{m,\nu }^4} \left[ \frac{\nu [\nu +1]}{\lambda _{m,\nu }^2} -1 \right] \,. \end{aligned}$$

### Lommel integral

The Lommel integral can be obtained from the general expression given in Eq. (35), respecting the Neumann boundary conditions, the Wronski-determinant from Eq. (97) and expression (98):

103 $$\begin{aligned}&\frac{1}{R^{\alpha + 1 }} \! \int \limits _{R}^{\gamma R} \!\! \mathrm {d}r\, r^{\alpha } \psi _{m,\nu }(r) \nonumber \\&= \frac{\gamma ^{-\frac{3}{2}}}{\lambda _{m,\nu }^{\frac{5}{2}+\alpha }} \frac{j_{\nu }^{\prime }(\lambda _{m,\nu })}{j_{\nu }^{\prime }(\gamma \lambda _{m,\nu })} \left[ \gamma \lambda _{m,\nu } [\alpha -\nu -2] S_{\alpha -\frac{3}{2},\nu +\frac{3}{2}}(\gamma \lambda _{m,\nu }) + \nu S_{\alpha -\frac{1}{2},\nu +\frac{1}{2}}(\gamma \lambda _{m,\nu }) \right] \nonumber \\&\quad - \frac{1}{\lambda _{m,\nu }^{\frac{5}{2}+\alpha }} \left[ \lambda _{m,\nu } [\alpha -\nu -2] S_{\alpha -\frac{3}{2},\nu +\frac{3}{2}}(\lambda _{m,\nu }) + \nu S_{\alpha -\frac{1}{2},\nu +\frac{1}{2}}(\lambda _{m,\nu }) \right] \,. \end{aligned}$$

For $\alpha +\nu =2p+1$, the Lommel functions can be expressed in terms of Gegenbauer polynomials as above [see Eq. (22)].

In analogy to Eq. (70), the special case for $\alpha =2$ and $\nu =0$ can be determined, since in this case the prefactors of the Lommel functions in Eq. (103) vanish:

104 $$\begin{aligned} \int \limits _{R}^{\gamma R} \!\! \mathrm {d}r\, r^{2} \psi _{m,0}(r) = 0 \,. \end{aligned}$$

Therefore, the functions 1 and $\psi _{m,0}(r)$ are orthogonal. In analogy to the cylindrical case in Eq. (71) this result can be generalized by integrating the original spherical Bessel differential equation (72)

105 $$\begin{aligned} \int \limits _{R}^{\gamma R} \mathrm {d}r\, r^2\left[ \frac{\nu [\nu +1]}{r^2} - \frac{\lambda _{m,\nu }^2}{R^2} \right] \psi _{m,\nu } (r) = 0 \,. \end{aligned}$$

## Numerical implementation

### Cylindrical Bessel functions

The discretization scheme provided for the radial part of the two-dimension Laplace operator with Neumann boundary conditions is implemented in the following algorithm: 

In lines 1 and 2, the length of the discretization interval *h* and the discretized radius according to the scheme (59) is computed. In lines 3–9, the discretized form of radial part of the two-dimensional Laplace operator given in Eq. (60) is implemented and, in line 10, the diagonal matrix $\nu ^2 \mathrm {diag}(1/r_1^2,\ldots,1/r_p^2)$ represents the part $\nu ^2/r^2$ of the original cylindrical Bessel differential equation. Finally, in line 11, the eigenvalue equation (61) is solved and the eigenvalues are given in a list sorted in ascending order. The eigenvalue $\lambda _{n,\nu }$ can be obtained by the command 

### Spherical Bessel functions

In analogy to the implementation for cylindrical Bessel functions, the eigenvalues of the radial part of the three-dimensional discretization scheme according to Eq. (94) and (95) can be implemented as follows: 

Finally, the eigenvalue $\lambda _{m,\nu }$ of the matrix eigenvalue problem (95) can be obtained by the command 

## Application to the diffusion process around dipole fields

To visualize the applicability of the results obtained in the previous sections, the frequency autocorrelation function of diffusing spins with the local resonance frequency

106 $$\begin{aligned} \omega ({\mathbf {r}}) = \delta \omega f({\mathbf {r}}) \end{aligned}$$

is considered as an example. The function $f({\mathbf {r}})$ describes the shape of the local resonance frequency and $\delta \omega$ its strength. The same analysis has already been performed in Ziener et al. (2008) for the diffusion between two concentric cylinders (two-dimensional case) and between two concentric spheres (three-dimensional case). The frequency autocorrelation function can be used to analyze magnetic resonance pulse sequences and to determine properties of red blood cells as shown in Ziener et al. (2010).

However, using the explicit expressions for the normalization and the Lommel integral for linear combinations of cylindrical or spherical Bessel functions, respectively, it is possible to obtain considerably simpler expressions as will be demonstrated below.

The frequency autocorrelation function for spins diffusing in the volume *V* is defined as (Ziener et al. 2006)

107 $$\begin{aligned} K(t)&= \delta \omega ^2 \int \mathrm {d}^3 {\mathbf {r}} \int \mathrm {d}^3 {\mathbf {r}}_0 f({\mathbf {r}}) p({\mathbf {r}},{\mathbf {r}}_0,t) f({\mathbf {r}}_0) p({\mathbf {r}}_0)\end{aligned}$$

108 $$\begin{aligned}&= \frac{\delta \omega ^2}{V} \int \mathrm {d}^3 {\mathbf {r}} \int \mathrm {d}^3 {\mathbf {r}}_0 f({\mathbf {r}}) p({\mathbf {r}},{\mathbf {r}}_0,t) f({\mathbf {r}}_0) \end{aligned}$$

where $p({\mathbf {r}}, {\mathbf {r}}_0,t)$ is the probability of a spin to diffuse during the time span *t* from the position ${\mathbf {r}}_0$ to the position ${\mathbf {r}}$ and $p({\mathbf {r}}_0) = 1/V$ is the probability of finding a spin at position ${\mathbf {r}}_0$. This transition probability is a solution of the diffusion equation

109 $$\begin{aligned} \frac{\partial }{\partial t} p({\mathbf {r}},{\mathbf {r}}_0,t) = D \Delta p({\mathbf {r}},{\mathbf {r}}_0,t) \end{aligned}$$

where *D* is the diffusion coefficient inside the volume *V* in which the diffusion occurs (Bauer et al. 2005). The transition probability $p({\mathbf {r}},{\mathbf {r}}_0,t)$ can be expressed in terms of an eigenfunction expansion

110 $$\begin{aligned} p({\mathbf {r}},{\mathbf {r}}_0,t) = \sum _{\kappa _l \ge 0}^{\infty } \mathrm {e}^{-\kappa _l^2 \frac{Dt}{R^2}} \phi _l({\mathbf {r}}) \phi _l^*({\mathbf {r}}_0) = \frac{1}{V} + \sum _{\kappa _l > 0}^{\infty } \mathrm {e}^{-\kappa _l^2 \frac{Dt}{R^2}} \phi _l({\mathbf {r}}) \phi _l^*({\mathbf {r}}_0) \end{aligned}$$

with eigenvalues $\kappa _l$ and eigenfunctions $\phi _l$ that fulfil the orthogonality condition

111 $$\begin{aligned} \int \mathrm {d}^3 {\mathbf {r}} \phi _l({\mathbf {r}}) \phi _{l^{\prime }}^*({\mathbf {r}}) = \delta _{ll^{\prime }} \end{aligned}$$

and are solutions of the eigenvalue equation

112 $$\begin{aligned} \Delta \phi _l({\mathbf {r}}) = - \frac{\kappa _l^2}{R^2} \phi _l({\mathbf {r}}) \,, \end{aligned}$$

with *R* being the radius of the inner cylinder or the inner sphere, respectively. The eigenfunction

113 $$\begin{aligned} \phi _0({\mathbf {r}}) = \frac{1}{\sqrt{V}} \end{aligned}$$

corresponds to the lowest eigenvalue

114 $$\begin{aligned} \kappa _0 = 0 \,. \end{aligned}$$

### Cylinders

In the two-dimensional case, the diffusion occurs in the space between two concentric circles with radius *R* and $\gamma R$:

115 $$\begin{aligned} V=\pi R^2 \left[ \gamma ^2-1 \right] \,. \end{aligned}$$

The eigenfunctions of the eigenvalue equation (112) are

116 $$\begin{aligned} \phi _{n,\nu }(r,\phi )&= \frac{\Phi _{n,\nu }(r)}{R\sqrt{N_{n,\nu }}} \frac{\mathrm {e}^{+\mathrm {i}\nu \phi }}{\sqrt{2\pi }} \end{aligned}$$

117 $$\begin{aligned} n&=+1,\ldots ,+\infty \end{aligned}$$

118 $$\begin{aligned} \nu&=-\infty , \ldots ,-1,0,+1,\ldots ,+\infty \,, \end{aligned}$$

with the radial eigenfunctions $\Phi _{n,\nu }(r)$ given in Eq. (39) and the according normalization constants $N_{n,\nu }$ given in Eq. (67) where the index $\nu$ takes the values $\nu =-\infty , \ldots ,-1,0,+1,\ldots ,+\infty$ and the index *n* lies in the range $n=+1,\ldots ,+\infty$. Using the symmetry relation of the radial eigenvalues in Eq. (45) and radial eigenfunctions in Eq. (47), the propagator from Eq. (110) can finally be written in the form

119 $$\begin{aligned} p(r,\phi ,r_0,\phi _0,t)&= \frac{1}{\pi R^2 \left[ \gamma ^2-1 \right] } \nonumber \\&\quad +\frac{1}{2\pi R^2} \sum _{n=1}^{\infty } \sum _{\nu =0}^{\infty } \frac{\mathrm {e}^{-\lambda _{n,\nu }^2 \frac{Dt}{R^2}}}{N_{n,\nu }} \Phi _{n,\nu }(r) \Phi _{n,\nu }(r_0) [2-\delta _{\nu 0}] \cos (\nu [\phi -\phi _0]) \,. \end{aligned}$$

**Fig. 5:**
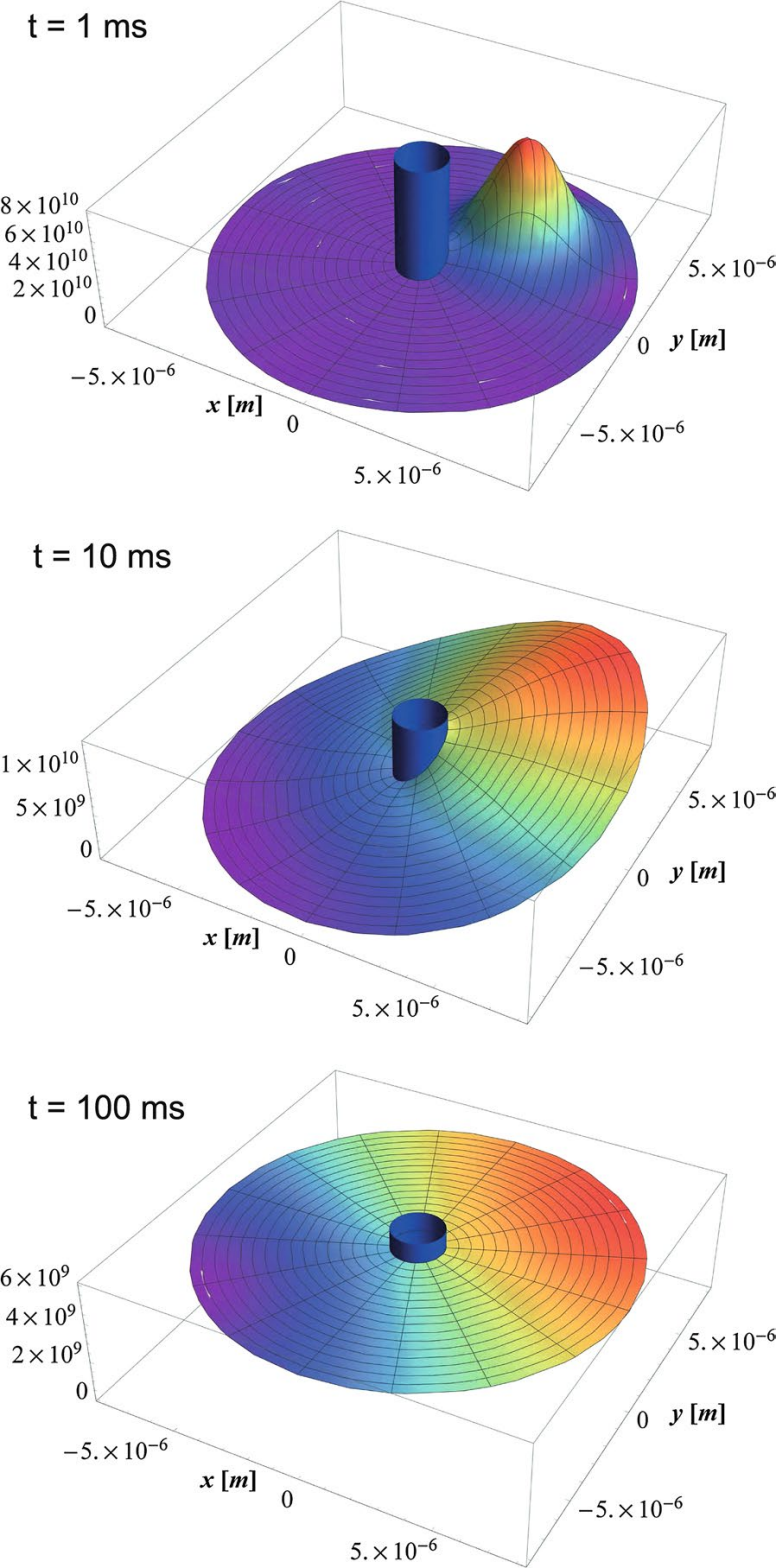
Diffusion propagator according to Eq. (119) around a cylinder with radius $R=1\,\mu \text {m}$, diffusion coefficient $D=1\, \mu \text {m}^2 / \text {ms}$ and $\gamma =8$. The initial position is at $r_0=4\,\mu \text {m}$ and $\phi _0=\pi /4$. For small times (exemplary for $t=1\,\text {ms}$) the transition probability is concentrated around the initial position. For large times (exemplary for $t=100\,\text {ms}$) the diffusion propagator takes the constant value $\lim _{t \rightarrow \infty } p(r,\phi ,r_0,\phi _0,t)=\frac{1}{\pi R^2 \left[ \gamma ^2-1 \right] } = 5.05254 \times 10^9 \,\text {m}^{-2}$.

In Fig. 5 the time evolution of this diffusion propagator is visualized.

Introducing the diffusion propagator and the shape-function of the two-dimensional dipole field

120 $$\begin{aligned} f(r,\phi ) = \frac{R^2}{r^2} \cos (2\phi ) \end{aligned}$$

into the definition of the autocorrelation function given in Eq. (108), only the addend with the index $\nu = 2$ remains and the autocorrelation function can finally be written in the form

121 $$\begin{aligned} K(t) = \delta \omega ^2 \sum _{n=1}^{\infty } F_n^2 \mathrm {e}^{- \lambda _{n,2}^2 \frac{Dt}{R^2}} \end{aligned}$$

with the expansion coefficients

122 $$\begin{aligned} F_n = \frac{1}{\sqrt{\gamma ^2-1}} \frac{1}{\sqrt{N_{n,2}}} \int \limits _{R}^{\gamma R} \mathrm {d} r \frac{\Phi _{n,2}(r)}{r} \,. \end{aligned}$$

A similar expression appears in Eq. (28) in Ziener et al. (2008) for the calculation of the expansion coefficients. The Lommel integral in Eq. (122) can be evaluated by using the general expression given in Eq. (69) with $\alpha =-1$ and $\nu =2$. In this special case the Lommel function can be expressed in terms of Schläfli polynomials [see Eq. (17) with $m=1$ and Eq. (21)]: $S_{-1,2}(z)=S_2(z)/4=1/z^2$, and, thus $S_{-1,2}^{\prime }(z) = -2/z^3$. Thus, one obtains:

123 $$\begin{aligned} F_n = \frac{1}{\sqrt{\gamma ^2-1}} \frac{1}{\sqrt{N_{n,2}}} \frac{4}{\pi \lambda _{n,2}^3} \left[ 1 - \frac{1}{\gamma ^3} \frac{J_{2}^{\prime }(\lambda _{n,2})}{J_{2}^{\prime }(\gamma \lambda _{n,2})} \right] \,. \end{aligned}$$

Finally, with the normalization constant given in Eq. (67), the expansion coefficient $F_n$ can be written as

124 $$\begin{aligned} F_n^2 = \frac{8}{\gamma ^2-1} \frac{1}{\lambda _{n,2}} \frac{\left[ 1-\frac{1}{\gamma ^3} \frac{J_{2}^{\prime }(\lambda _{n,2})}{J_{2}^{\prime }(\gamma \lambda _{n,2})}\right] ^2}{4-\lambda _{n,2}^2 + \left[ \lambda _{n,2}^2 - \frac{4}{\gamma ^2} \right] \left[ \frac{J_{2}^{\prime }(\lambda _{n,2})}{J_{2}^{\prime }(\gamma \lambda _{n,2})}\right] ^2} \end{aligned}$$

or

125 $$\begin{aligned} F_n^2 = \frac{8}{\gamma ^2-1} \frac{1}{\lambda _{n,2}^2} \frac{1}{\gamma ^4} \frac{\left[ \gamma ^3 J_{2}^{\prime }(\gamma \lambda _{n,2}) - J_{2}^{\prime }(\lambda _{n,2})\right] ^2}{\gamma ^2 \left[ 4-\lambda _{n,2}^2 \right] \left[ J_{2}^{\prime }(\gamma \lambda _{n,2}) \right] ^2 + \left[ \gamma ^2 \lambda _{n,2}^2 - 4 \right] \left[ J_{2}^{\prime }(\lambda _{n,2}) \right] ^2} \,. \end{aligned}$$

This expression for the expansion coefficients is evidently a simpler expression than its equivalent given in Eq. (B1) in Ziener et al. (2008).

The corresponding eigenvalues $\lambda _{n,2}$ can be obtained from Eq. (41) for $\nu =2$:

126 $$\begin{aligned} Y_{2}^{\prime }(\lambda _{n,2}) J_{2}^{\prime } (\gamma \lambda _{n,2}) = J_{2}^{\prime }(\lambda _{n,2}) Y_{2}^{\prime } (\gamma \lambda _{n,2}) \, , \end{aligned}$$

and the following sums can be used to check if a sufficient numerical accuracy is obtained:

127 $$\begin{aligned} \sum _{n=1}^{\infty } F_n^2&= \frac{1}{2 \gamma ^2} \end{aligned}$$

128 $$\begin{aligned} \sum _{n=1}^{\infty } F_n^2 \lambda _{n,2}^2&= 2\frac{1+\gamma ^2}{\gamma ^4} \end{aligned}$$

129 $$\begin{aligned} \sum _{n=1}^{\infty } \frac{F_n^2}{\lambda _{n,2}^2}&= \frac{\mathrm {ln}(\gamma )}{4[\gamma ^2-1]} \end{aligned}$$

130 $$\begin{aligned} \sum _{n=1}^{\infty } \frac{F_n^2}{\lambda _{n,2}^4}&= \frac{1}{32} \,. \end{aligned}$$

### Spheres

In the three-dimensional case the diffusion occurs between two concentric spheres, and, thus, the diffusion volume corresponds to

131 $$\begin{aligned} V=\frac{4}{3}\pi R^3 \left[ \gamma ^3 -1 \right] \,. \end{aligned}$$

The orthogonal eigenfunctions of the eigenvalue equation (112) are

132 $$\begin{aligned} \phi _{m,\nu ,\mu }(r,\theta ,\phi )&= \frac{\psi _{m,\nu }(r)}{\sqrt{R^3 N_{m,\nu }}} Y_{\nu ,\mu } (\theta ,\phi ) \end{aligned}$$

133 $$\begin{aligned} m&= +1, \ldots , +\infty \end{aligned}$$

134 $$\begin{aligned} \nu&= 0,+1,\ldots ,+\infty \end{aligned}$$

135 $$\begin{aligned} \mu&= - \nu , \ldots , 0 , \ldots , + \nu \,, \end{aligned}$$

with the radial eigenfunctions $\psi _{m,\nu }(r)$ given in Eq. (75), the respective normalization constants $N_{m,\nu }$ are given in Eq. (102) and the spherical harmonics are defined by

136 $$\begin{aligned} Y_{\nu ,\mu } (\theta ,\phi ) = \sqrt{\frac{2\nu +1}{4\pi } \frac{[\nu -\mu ]!}{[\nu +\mu ]!}} P_{\nu }^{\mu }(\cos (\theta )) \mathrm {e}^{\mathrm {i}\mu \phi } \,, \end{aligned}$$

where $P_{\nu }^{\mu }$ denote the associated Legendre polynomials which are defined for the negative upper index as

137 $$\begin{aligned} P_{\nu }^{-\mu } (\cos (\theta )) = [-1]^{\mu } \frac{[\nu -\mu ]!}{[\nu +\mu ]!} P_{\nu }^{\mu } (\cos (\theta )) \,. \end{aligned}$$

In contradiction to the two-dimensional case, the index $\nu$ in the three-dimensional case is in the range $\nu =0,+1,\ldots ,+\infty$. The index $\mu$ takes the values $\mu = - \nu , \ldots , 0 , \ldots , + \nu$. Therefore, the propagator given in Eq. (110) can be written as

138 $$\begin{aligned} p(r,\theta ,\phi ,r_0,\theta _0,\phi _0,t)&= \frac{3}{4\pi R^3 \left[ \gamma ^3-1 \right] } \nonumber \\& \quad +\frac{1}{R^3} \sum _{m=1}^{\infty } \sum _{\nu =0}^{\infty } \sum _{\mu =-\nu }^{+\nu } \frac{\mathrm {e}^{-\lambda _{m,\nu }^2 \frac{Dt}{R^2}}}{N_{m,\nu }} \psi _{m,\nu }(r) \psi _{m,\nu }(r_0) Y_{\nu ,\mu } (\theta ,\phi ) Y_{\nu ,\mu }^{*} (\theta _0,\phi _0) \,. \end{aligned}$$

The sum over the index $\mu$ can further be simplified by using the explicit expression of the spherical harmonics given in Eq. (136) and the definition of the associated Legendre polynomials with a negative upper index from Eq. (137):

139 $$\begin{aligned} \sum _{\mu =-\nu }^{+\nu } Y_{\nu ,\mu } (\theta ,\phi ) Y_{\nu ,\mu }^{*} (\theta _0,\phi _0)&= \frac{2 \nu + 1}{4\pi } P_{\nu } \left( \cos (\theta ) \right) P_{\nu } \left( \cos (\theta _0) \right) \nonumber \\&\quad + \frac{2 \nu + 1}{2\pi } \sum _{\mu =+1}^{+\nu } \frac{[\nu -\mu ]!}{[\nu +\mu ]!} P_{\nu }^{\mu } (\cos (\theta )) P_{\nu }^{\mu } (\cos (\theta _0)) \cos \left( \mu [\phi -\phi _0] \right) \nonumber \\&= \frac{2 \nu + 1}{4\pi } \sum _{\mu =0}^{+\nu } \frac{[\nu -\mu ]!}{[\nu +\mu ]!} P_{\nu }^{\mu } (\cos (\theta )) P_{\nu }^{\mu } (\cos (\theta _0)) [2-\delta _{\mu 0}]\cos \left( \mu [\phi -\phi _0] \right) \,. \end{aligned}$$

Evidently, the transition probability $p(r,\theta ,\phi ,r_0,\theta _0,\phi _0,t)$ to go in time *t* from position $(r_0,\theta _0,\phi _0)$ to position $(r,\theta ,\phi )$ depends on the radial distances $r_0$ and *r* and the spherical angle $\Omega = (\theta _0,\phi _0;\theta ,\phi )$ that can be determined by the spherical law of cosines:

140 $$\begin{aligned} \cos (\Omega )=\cos (\theta _0) \cos (\theta ) + \sin (\theta _0) \sin (\theta ) \cos (\phi _0 - \phi ) \,. \end{aligned}$$

Using the addition theorem for spherical harmonics [see 14.30.9 in Olver et al. (2010)]

141 $$\begin{aligned} \sum _{\mu =-\nu }^{+\nu } Y_{\nu ,\mu } (\theta ,\phi ) Y_{\nu ,\mu }^{*} (\theta _0,\phi _0)&= \frac{2 \nu + 1}{4\pi } P_{\nu } \left( \cos (\theta _0) \cos (\theta ) + \sin (\theta _0) \sin (\theta ) \cos (\phi _0 - \phi )\right) \nonumber \\&= \frac{2 \nu + 1}{4\pi } P_{\nu } \left( \cos (\Omega ) \right) \end{aligned}$$

the transition probability can finally be written in the form

142 $$\begin{aligned} p(r,r_0,\Omega ,t) = \frac{1}{4\pi R^3} \left[ \frac{3}{\gamma ^3-1} + \sum _{m=1}^{\infty } \sum _{\nu =0}^{\infty } \frac{2\nu + 1}{N_{m,\nu }} \mathrm {e}^{-\lambda _{m,\nu }^2 \frac{Dt}{R^2}} \psi _{m,\nu }(r) \psi _{m,\nu }(r_0) P_{\nu } (\cos (\Omega )) \right] \,. \end{aligned}$$

Comparing the addition theorem for spherical harmonics in Eq. (141) with the sum over the product of the spherical harmonics in Eq. (139), the results of Eq. (10) on page 382 of section 14.16. I. in Carslaw and Jaeger (1959) can be reproduced.

The three-dimensional dipole field has the shape

143 $$\begin{aligned} f(r,\theta ,\phi ) = \frac{R^3}{r^3} \left[ 3\cos ^2(\theta )-1\right] = 4 \sqrt{\frac{\pi }{5}} Y_{20}(\theta ,\phi ) \frac{R^3}{r^3} \,. \end{aligned}$$

Introducing this shape function and the three-dimensional propagator from Eq. (138) into the definition of the autocorrelation function (108), leads to

144 $$\begin{aligned} K(t) = \delta \omega ^2 \sum _{m=1}^{\infty } F_m^2 \mathrm {e}^{- \lambda _{m,2}^2 \frac{Dt}{R^2}} \end{aligned}$$

with the expansion coefficients

145 $$\begin{aligned} F_m&= \sqrt{\frac{3}{4 \pi R^3 \left[ \gamma ^3 -1 \right] }} \int \limits _{0}^{\pi }\!\!\! \mathrm {d} \theta \!\! \int \limits _{0}^{2\pi }\!\!\! \mathrm {d} \phi \!\! \int \limits _{R}^{\gamma R} \!\!\! \mathrm {d} r\, r^2 \sin (\theta ) 4\! \sqrt{\frac{\pi }{5}} Y_{20}(\theta ,\phi ) \frac{R^3}{r^3} \frac{Y_{20}(\theta ,\phi )}{\sqrt{R^3 N_{m,2}}} \psi _{m,2}(r) \end{aligned}$$

146 $$\begin{aligned}= \frac{1}{\sqrt{\gamma ^3-1}} \sqrt{\frac{3}{5}} \frac{2}{\sqrt{N_{m,2}}} \int \limits _{R}^{\gamma R} \mathrm {d} r \frac{\psi _{m,2}(r)}{r} \,. \end{aligned}$$

The remaining Lommel integral can be solved by using the expression (103) for $\alpha =-1$ and $\nu =2$. The occurring Lommel functions $S_{-\frac{5}{2},+\frac{7}{2}}(z)$ and $S_{-\frac{3}{2},+\frac{5}{2}}(z)$ can explicitly be given by using the relation (22):

147 $$\begin{aligned} S_{-\frac{5}{2},+\frac{7}{2}}(z)&= \frac{2}{7} \frac{z^{-\frac{5}{2}}}{2^{\frac{7}{2}} \Gamma (\frac{7}{2})} A_{0,\frac{7}{2}}(z) = z^{-\frac{7}{2}}\end{aligned}$$

148 $$\begin{aligned} S_{-\frac{3}{2},+\frac{5}{2}}(z)&= \frac{2}{5} \frac{z^{-\frac{3}{2}}}{2^{\frac{5}{2}} \Gamma (\frac{5}{2})} A_{0,\frac{5}{2}}(z) = z^{-\frac{5}{2}} \end{aligned}$$

where $A_{0,\frac{7}{2}}(z)$ and $A_{0,\frac{5}{2}}(z)$ can be obtained from Eq. (25). Thus, the integration yields:

149 $$\begin{aligned} F_m = \frac{1}{\sqrt{\gamma ^3-1}} \sqrt{\frac{3}{5}} \frac{6}{\sqrt{N_{m,2}}} \frac{1}{\lambda _{m,2}^4} \left[ 1- \frac{1}{\gamma ^4} \frac{j_{2}^{\prime }(\lambda _{m,2})}{j_{2}^{\prime }(\gamma \lambda _{m,2})} \right] \,. \end{aligned}$$

Introducing the normalization constant from Eq. (102) yields:

150 $$\begin{aligned} F_m^2 = \frac{216}{5} \frac{1}{\lambda _{m,2}^2} \frac{1}{\gamma ^3-1} \frac{\left[ 1 - \frac{1}{\gamma ^4} \frac{j_{2}^{\prime }(\lambda _{m,2})}{j_{2}^{\prime }(\gamma \lambda _{m,2})} \right] ^2}{\frac{\gamma ^2 \lambda _{m,2}^2-6}{\gamma ^3}\left[ \frac{j_{2}^{\prime }(\lambda _{m,2})}{j_{2}^{\prime }(\gamma \lambda _{m,2})} \right] ^2 +6-\lambda _{m,2}^2} \,. \end{aligned}$$

For $\nu =2$ the spherical Bessel functions can be expressed in terms of the sine and cosine function:

151 $$\begin{aligned} j_2(z)&= \left[ \frac{3}{z^3} - \frac{1}{z} \right] \sin (z) - \frac{3}{z^2} \cos (z) \end{aligned}$$

152 $$\begin{aligned} y_2(z)&= - \left[ \frac{3}{z^3} - \frac{1}{z} \right] \cos (z) - \frac{3}{z^2} \sin (z) \end{aligned}$$

which will be necessary for further simplifications. The derivative of the spherical Bessel functions can be simplified by using the relation (151):

153 $$\begin{aligned} \frac{j_{2}^{\prime }(\lambda _{m,2})}{j_{2}^{\prime }(\gamma \lambda _{m,2})} = \gamma ^4 \frac{[4\lambda _{m,2}^2-9]\sin (\lambda _{m,2})+ \lambda _{m,2}[9-\lambda _{m,2}^2]\cos (\lambda _{m,2})}{[4\gamma ^2 \lambda _{m,2}^2 - 9] \sin (\gamma \lambda _{m,2})+ \gamma \lambda _{m,2} [9-\gamma ^2 \lambda _{m,2}^2]\cos (\gamma \lambda _{m,2})} \,. \end{aligned}$$

Finally, the expansion coefficients can be written as

154 $$\begin{aligned} F_m^2 = \frac{216}{5} \frac{1}{\lambda _{m,2}^2} \frac{1}{\gamma ^3-1} \frac{\left[ 1 - \frac{[4 \lambda _{m,2}^2-9]\sin (\lambda _{m,2})+ \lambda _{m,2}[9-\lambda _{m,2}^2]\cos (\lambda _{m,2})}{[4\gamma ^2 \lambda _{m,2}^2-9]\sin (\gamma \lambda _{m,2})+ \gamma \lambda _{m,2}[9-\gamma ^2 \lambda _{m,2}^2]\cos (\gamma \lambda _{m,2})} \right] ^2}{\gamma ^5 \left[ \frac{[4\lambda _{m,2}^2-9]\sin (\lambda _{m,2})+ \lambda _{m,2}[9-\lambda _{m,2}^2]\cos (\lambda _{m,2})}{[4\gamma ^2\lambda _{m,2}^2-9]\sin (\gamma \lambda _{m,2})+ \gamma \lambda _{m,2}[9-\gamma ^2\lambda _{m,2}^2]\cos (\gamma \lambda _{m,2})}\right] ^2 \left[ \gamma ^2\lambda _{m,2}^2-6\right] +6-\lambda _{m,2}^2} \end{aligned}$$

or in the form

155 $$\begin{aligned}&\textstyle {F_m^2=\frac{216}{5} \frac{1}{\lambda _{m,2}^2} \frac{1}{\gamma ^3-1}} \nonumber \\& \times \textstyle { \frac{\left[ [4\gamma ^2\lambda _{m,2}^2-9]\sin (\gamma \lambda _{m,2})+ \gamma \lambda _{m,2} [9-\gamma ^2\lambda _{m,2}^2 ]\cos (\gamma \lambda _{m,2}) - [4\lambda _{m,2}^2-9 ]\sin (\lambda _{m,2}) - \lambda _{m,2} [9-\lambda _{m,2}^2 ]\cos (\lambda _{m,2}) \right] ^2}{\gamma ^5 \left[ [4 \lambda _{m,2}^2-9 ]\sin (\lambda _{m,2})+ \lambda _{m,2} [9-\lambda _{m,2}^2 ]\cos (\lambda _{m,2}) \right] ^2 [\gamma ^2 \lambda _{m,2}^2 - 6 ]+ [6-\lambda _{m,2}^2 ] \left[ [ 4\gamma ^2\lambda _{m,2}^2 - 9 ] \sin (\gamma \lambda _{m,2})+ \gamma \lambda _{m,2} [ 9 - \gamma ^2 \lambda _{m,2}^2 ] \cos (\gamma \lambda _{m,2}) \right] ^2}} \,. \end{aligned}$$

This expression for the expansion coefficients is much more simpler than the equivalent expression given in Eq. (B4) in Ziener et al. (2008) or in Eq. (7) in Ziener et al. (2010).

The eigenvalues can be obtained from Eq. (77) for $\nu =2$, which can be further simplified using the expressions (151) and (152). Finally, the following transcendental equation has to be solved:

156 $$\begin{aligned} \frac{\tan (\lambda _{m,2} [\gamma -1])}{\lambda _{m,2} [\gamma -1]} = \frac{81 - 9 \lambda _{m,2}^2 [\gamma ^2 - 3 \gamma +1] + 4 \gamma ^2 \lambda _{m,2}^4}{81-9 \lambda _{m,2}^2 [4\gamma ^2 - 9 \gamma +4 ] + \gamma \lambda _{m,2}^4 [16 \gamma - 9 \gamma ^2 -9] + \gamma ^3 \lambda _{m,2}^6} \,. \end{aligned}$$

This equation coincides with Eq. (5) in Kurz et al. (2014) for $\lambda _{m,2}=\sqrt{\kappa }$ and $\gamma = \eta ^{-1/3}$.

The following sums can be used to check if a sufficient numerical accuracy is obtained:

157 $$\begin{aligned} \sum _{m=1}^{\infty } F_m^2&= \frac{4}{5 \gamma ^3} \end{aligned}$$

158 $$\begin{aligned} \sum _{m=1}^{\infty } F_m^2 \lambda _{m,2}^2&= \frac{36}{5\gamma ^5} \frac{\gamma ^5-1}{\gamma ^3-1} \end{aligned}$$

159 $$\begin{aligned} \sum _{m=1}^{\infty } \frac{F_m^2}{\lambda _{m,2}^2}&= \frac{2}{5} \frac{1}{\gamma ^3-1} \left[ 1-\frac{1}{\gamma }+\frac{4[\gamma ^3 - 1]^2 + 9 \gamma [2\gamma ^2 - 1 - \gamma ^4]}{36\gamma [1-\gamma ^5]}\right] \,. \end{aligned}$$

## Summary and conclusions

Cylindrical and spherical Bessel functions are frequently employed in treating boundary value and eigenvalue problems in applied mathematics (Gray and Mathews 1895; McLachlan 1941; Kac 1966). In this work, the general orthogonality relation and Lommel integral of a linear combination of both cylindrical and spherical Bessel functions, respectively, are considered. For a given radius interval of $R \le r \le \gamma R$, Neumann boundary conditions are assumed and the corresponding approximated first eigenvalues $\lambda _{1,\nu }$ for small $\lambda$ and $\nu$ are compared in their $\gamma$-dependency with expressions from the literature in the same limit and the exact numerical solution for cylindrical and spherical Bessel functions, respectively (see Figs. 3, 4). It is shown that all expressions agree well for $\gamma \rightarrow 1$. Consequently, explicit terms for the orthogonality relation, normalization constant and Lommel integral are given in both model geometries using a discretized form of the respective Bessel differential equations. In the case of spherical Bessel functions, these results are a generalization from integer indices, that can be presented in terms of trigonometric functions and have so mostly to treat related physical problems (Carslaw and Jaeger 1959), to arbitrary complex-valued indices. Moreover, a numerical implementation to calculate the relevant eigenvalues is provided and the results are applied to a scenario in magnetic resonance physics where spin diffusion processes between two magnetic concentric cylinders or two concentric spheres occur. Thereby, an eigenfunction expansion of the spin transition probability function results in analytic expressions for the expansion coefficients of the frequency autocorrelation function *K*(*t*). These are mathematically easier to grasp and, therefore, to implement numerically, than equivalent expressions that have been derived recently (Ziener et al. 2008). The results might be useful to obtain analytical expressions for the transverse relaxation in Carr-Purcell-Meiboom-Gill experiments where Jensen and Chandra have provided a relation between the diffusion-dependent part of transverse relaxation and the frequency autocorrelation function within a weak field approximation (Jensen et al. 2001; Jensen and Chandra 2000). With the paramagnetic properties of deoxygenated hemoglobin, such analyses may then be applied to study highly organized (cylindriform) capillary arrangements in, for instance, skeletal muscle tissue (Ziener et al. 2012). Likewise, the results for spherical geometries may be used to quantify microstructural parameters in lung tissue by treating lung alveoli as spherical entities that are surrounded by a dense capillary network. A further analysis might consider mixed boundary conditions and/or extend the results to other model geometries.
